# Body size in early life and risk of epithelial ovarian cancer: results from the Nurses' Health Studies

**DOI:** 10.1038/sj.bjc.6604742

**Published:** 2008-10-28

**Authors:** H J Baer, S E Hankinson, S S Tworoger

**Affiliations:** 1 Division of General Medicine, Department of Medicine, Brigham and Women's Hospital and Harvard Medical School, Boston, MA, USA; 2 Department of Epidemiology, Harvard School of Public Health, Boston, MA, USA; 3 Channing Laboratory, Department of Medicine, Brigham and Women's Hospital and Harvard Medical School, Boston, MA, USA

**Keywords:** childhood, body mass index, birthweight, height, ovarian cancer

## Abstract

Adult body mass index (BMI) has been associated with ovarian cancer risk, but few studies have examined body size earlier in life. We prospectively examined associations of body fatness at ages 5 and 10, BMI at age 18, height, and birthweight with risk of epithelial ovarian cancer in the Nurses' Health Study (NHS: 110 311 women, 735 cases) and Nurses' Health Study II (NHSII: 113 059 women, 137 cases). Cox proportional hazards regression was used to estimate relative risks (RRs) and 95% confidence intervals (CIs). There was a weak inverse association between average body fatness at ages 5 and 10 and risk in the NHS (RR for heaviest *vs* most lean=0.81, 95% CI: 0.53–1.24, *P* for trend=0.04) and a nonsignificant positive association in the NHSII (RR=2.09, 95% CI: 0.98–4.48, *P* for trend=0.10), possibly due to differences in age and menopausal status. Height was positively associated with risk in both cohorts (RR for ⩾1.75 *vs* <1.6 m=1.43, 95% CI: 1.05–1.96, *P* for trend=0.001). Body mass index at the age of 18 years and birthweight were not associated with risk. Further research should examine the biological mechanisms underlying the observed associations.

Overweight and obesity have been associated with risk of cancer in women (Calle and Kaaks, 2004), but the findings for ovarian cancer are inconclusive. Some epidemiological studies have observed weak to moderate positive associations between adult body mass index (BMI) and ovarian cancer risk; others have found no association (Olsen *et al*, 2007). One potential explanation is that the timing of overweight and obesity during the lifecourse may be important. Although most studies have examined ovarian cancer risk in relation to recent BMI, studies examining BMI earlier in life, during late adolescence or young adulthood, have observed stronger positive associations (Fairfield *et al*, 2002; Engeland *et al*, 2003; Lubin *et al*, 2003; Anderson *et al*, 2004). Body fatness during childhood and early adolescence has been associated with breast cancer risk, independent of adult BMI (Berkey *et al*, 1999; Baer *et al*, 2005), suggesting that adiposity at young ages may affect risk of hormone-related cancers. Furthermore, there is evidence that height, a marker of early childhood growth and nutrition, is associated with ovarian cancer risk (Rodriguez *et al*, 2002; Engeland *et al*, 2003; Schouten *et al*, 2003, 2008), indicating that early life may be a critical time period for ovarian cancer initiation.

Therefore, we prospectively examined the associations of body size in early life – including body fatness at ages 5 and 10 years, BMI at the age of 18 years, birthweight, and height – with risk of epithelial ovarian cancer among participants in two large cohort studies, the Nurses' Health Study (NHS) and the NHSII.

## Materials and methods

### Study design and population

The NHS began in 1976 and the NHSII in 1989, when 121 700 and 116 609 US female registered nurses, respectively, completed a mailed questionnaire about their lifestyle factors, health behaviours, and medical histories. Follow-up questionnaires have been sent to participants every 2 years since enrolment. Incident cases of epithelial ovarian cancer were reported on the biennial questionnaires through 2004 (NHS) and 2005 (NHSII). A gynaecologic pathologist reviewed the pathology reports and medical records to confirm the diagnosis and identify histological type, subtype, morphology, and stage (Tworoger *et al*, 2008).

### Assessment of body size in early life and other covariates

**Figure 1:**
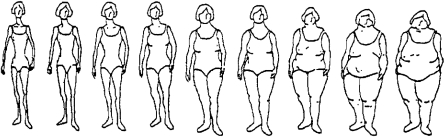
Figure drawing used to assess body fatness at ages 5 and 10 years.

Participants recalled their body fatness (also called ‘somatotype’) at ages 5 and 10 years using a nine-level figure drawing, where level 1 represents the most lean and level 9 represents the most overweight (Figure 1) (Stunkard *et al*, 1983). Among participants in the Third Harvard Growth Study, Pearsons correlations between recalled somatotype and measured BMI were 0.60 for the age of 5 years and 0.70 for the age of 10 years (Must *et al*, 1993). We averaged each participant's reported somatotypes at ages 5 and 10 years to obtain an estimate of childhood body fatness. The levels 5 and above were combined in the analysis because of small numbers of participants in these categories.

Women reported their weight at the age of 18 years and their current height at enrolment; these were used to calculate BMI at the age of 18 years in kg m^−2^. In a sample of NHSII participants, the Spearmans correlation between recalled and recorded weight at the age of 18 years was 0.87, and for BMI at the age of 18 years was 0.84 (Troy *et al*, 1995). Categories for BMI at the age of 18 years and height were chosen based on their distributions and previously used cutpoints.

Participants recalled their birthweight as <5.5, 5.5–6.9, 7.0–8.4, 8.5–9.9, and ⩾10 pounds. The two highest categories (8.5 pounds and higher) were combined in the analysis to increase power. In a NHSII validation study (Troy *et al*, 1996), the correlation between self-reported birthweight and that obtained from state birth records was 0.74.

Age and other covariates were assessed on the questionnaires throughout the study.

### Statistical analysis

Participants contributed person-time from baseline (the questionnaire year that the exposure of interest was assessed) until the date of ovarian cancer diagnosis, report of other cancer (except nonmelanoma skin cancer), death, or 31 May 2004 (NHS) or 31 May 2005 (NHSII), whichever occurred sooner. We excluded women reporting a previous diagnosis of cancer except nonmelanoma skin cancer and those with a history of bilateral oophorectomy or pelvic irradiation. For analyses focusing on each individual body size measure, we excluded women who were missing data for that measure. The years of assessment, numbers of eligible participants and cases, and total numbers of person-years available for each analysis are shown in Table 1.

Cox proportional hazards models stratified by age in months and 2-year questionnaire cycle were used to estimate relative risks (RRs) and 95% confidence intervals (CIs), adjusting for ovarian cancer risk factors. Tests for linear trend were conducted by including each body size measure in a model either as an ordinal variable (childhood body fatness, birthweight) or as a continuous variable with values equal to the category medians (BMI at the age of 18 years, height).

We first conducted the analyses within the NHS and NHSII separately. We then evaluated heterogeneity in the estimates by cohort (DerSimonian and Laird, 1986). We used interaction terms and stratified analyses to assess effect modification by menopausal status, age, and common ovarian cancer risk factors. Separate models were run for invasive cases alone and by histological type (serous/poorly-differentiated, endometrioid, mucinous).

## Results

Most adult characteristics were not associated with early life body size (Table 2). In both cohorts, greater body fatness during childhood and BMI at the age of 18 years were associated with earlier menarche and higher current BMI, and taller height was associated with later menarche. In the NHS, women with greater childhood body fatness and taller women were slightly younger at baseline, and women who were heavier in childhood and at the age of 18 years were slightly less likely to use postmenopausal hormones. In the NHSII, women with greater childhood body fatness, greater BMI at the age of 18 years, and taller height were less likely to be parous, and those with greater BMI at the age of 18 years also had shorter duration of oral contraceptive use.

In the NHS, greater body fatness at ages 5 and 10 years were associated with decreased risk of ovarian cancer (Table 3), although the association was only significant for the age of 10 years (RR for level ⩾5 *vs* level 1=0.69, 95% CI: 0.48–0.99, *P* for trend=0.01). Averaging ages 5 and 10 years, the RR for childhood body fatness level ⩾5 compared to level 1 was 0.81 (95% CI: 0.53–1.24, *P* for trend=0.04). In contrast, there was some suggestion of a positive association for average childhood body fatness in the NHSII (RR for level ⩾5 *vs* level 1=2.09, 95% CI: 0.98–4.48), although this was not statistically significant (*P* for trend=0.10) (Table 3). The associations for body fatness at ages 5 and 10 years individually and average childhood body fatness were significantly different by cohort (*P* for heterogeneity=0.03, 0.01, and 0.01, respectively). Body mass index at the age of 18 years was not significantly associated with risk in either cohort.

One major difference between the NHS and NHSII cohorts is the menopausal status of participants when childhood body size was assessed (NHS: 32% premenopausal in 1988, NHSII: 99% premenopausal in 1989). To explore whether this could explain the observed difference in the association of childhood body fatness with risk, we combined the data from both cohorts and stratified by menopausal status in the cycle before diagnosis (Table 4). There was some suggestion of a weak positive association between childhood body fatness and risk of ovarian cancer in premenopausal women (pooled RR for level ⩾5 *vs* level 1=1.38, 95% CI: 0.70–2.71, *P* for trend=0.92) and a weak inverse association in postmenopausal women (comparable RR=0.85, 95% CI: 0.54–1.31, *P* for trend=0.09), although neither these nor the interaction with menopausal status (*P*=0.37) were statistically significant. Results for BMI at the age of 18 years were similar in premenopausal women, but there was no evidence of an inverse association in postmenopausal women (*P* for interaction=0.11).

Alternatively, the observed variation in the associations for childhood body fatness could be explained by age differences between participants in the two cohorts (NHS: mean age=54.3 in 1988, NHSII: mean age=34.3 in 1989); therefore, we conducted a preliminary analysis combining both cohorts and stratifying by age, while adjusting for menopausal status. There were nonsignificant positive associations between childhood body fatness and ovarian cancer risk in women less than the age of 50 years (pooled RR for level ⩾5 *vs* level 1=1.77, 95% CI: 0.85–3.69, *P* for trend=0.39) and in women between the ages 50 and 59 years (comparable RR=1.30, 95% CI: 0.64–2.65, *P* for trend=0.34), whereas the association was inverse in women at the age of 60 years and older (comparable RR=0.67, 95% CI: 0.39–1.16, *P* for trend=0.01). The interaction between childhood body fatness and age was statistically significant (*P*=0.001). When we jointly stratified by age and menopausal status, the positive association between childhood body fatness and ovarian cancer risk appeared stronger in premenopausal women under the age of 45 years (pooled RR for childhood body fatness level ⩾5 *vs* level 1=2.51, 95% CI: 0.94–6.73) than in those at the age of 45 years and older (comparable RR=0.83, 95% CI: 0.30–2.28). Conversely, the inverse association in postmenopausal women was stronger among those at the age of 60 years and older (pooled RR for childhood body fatness level ⩾5 *vs* level 1=0.67, 95% CI: 0.39–1.16) than in those younger than the age of 60 years (comparable RR=1.63, 95% CI: 0.73–3.66).

Additional adjustment for current BMI as a continuous variable had virtually no impact on the childhood body fatness associations in the NHS or for postmenopausal women overall (data not shown). However, the positive association for childhood body fatness in the NHSII and in premenopausal women was attenuated when including current BMI (RR for childhood body fatness level ⩾5 *vs* level 1 in premenopausal women=1.23, 95% CI: 0.61–2.47, *P* for trend=0.73). Adjustment for age at menarche had no substantial impact on the associations (data not shown).

Height was positively associated with ovarian cancer risk in both cohorts (pooled RR for ⩾1.75 *vs* <1.6 m=1.43, 95% CI: 1.05–1.96, *P* for trend=0.001), and the test for heterogeneity was not significant (*P*=0.22); however, the association appeared stronger in the NHSII (comparable RR=2.35, 95% CI: 1.19–4.63, *P* for trend=0.01) than in the NHS (comparable RR=1.27, 95% CI: 0.88–1.82, *P* for trend=0.01) (Table 5). The positive association was slightly stronger among premenopausal than postmenopausal women, although there were no significant interactions with menopausal status or age (data not shown). Birthweight was not significantly associated with risk of ovarian cancer in either cohort.

The observed associations for body size in early life were similar for invasive cases alone and by histological type, although these analyses were limited by small case numbers. No significant interactions were observed between any of the body size measures and parity, oral contraceptive use, postmenopausal hormone use, or family history of breast or ovarian cancer (data not shown).

## Discussion

Results from this study indicate that body size in early life may be related to the risk of epithelial ovarian cancer. Body fatness during childhood was associated with ovarian cancer risk, although the association differed by cohort; greater body fatness at ages 5 and 10 years was associated with a slightly lower risk among NHS women, but a suggestion of an increased risk among NHSII women. These differences could be explained by differences in the menopausal status or age of participants. Body mass index at the age of 18 years and birthweight were not associated with risk; however, height was positively associated with risk.

To our knowledge, this is the first study to investigate the association of childhood fatness with ovarian cancer risk. Previous studies have examined the relation between overweight and obesity in adulthood and ovarian cancer risk or mortality, with mixed results (Olsen *et al*, 2007). One possible reason for these inconsistencies pertains to the timing of body size assessment. Several studies have observed stronger associations for BMI in adolescence or young adulthood than for recent BMI (Engeland *et al*, 2003; Anderson *et al*, 2004). The results from our study indicate that body size at even younger ages may be an important predictor of ovarian cancer risk.

A second potential explanation is that the relation between body size at young ages and ovarian cancer risk may differ by menopausal status or age. In a pooled analysis of 12 cohorts, BMI at baseline was not associated with risk overall or among postmenopausal women, but there was a positive association in premenopausal women (Schouten *et al*, 2008). Our findings also suggest that the positive association for childhood body fatness may be limited to premenopausal women, particularly younger premenopausal women.

However, our findings of different associations for childhood body fatness with ovarian cancer risk by cohort could be partially due to a cohort effect. Greater childhood body fatness among women in the NHS may have been determined by different factors than in the NHSII. Chance could also explain our results, given that the observed associations for childhood body fatness were modest and the trends were only marginally significant. However, strong associations between childhood body fatness and risk of breast cancer have been observed in previous studies, suggesting that it may predict the risk of hormone-related cancers in women. Further, previous studies have observed significant associations of both adolescent and adult BMI with ovarian cancer risk, lending plausibility to our findings.

The associations of body size in childhood with ovarian cancer risk could be mediated through endogenous hormones (Risch, 1998; Lukanova and Kaaks, 2005), although epidemiological data on the relation of endogenous hormones with ovarian cancer risk are sparse and inconsistent (Eliassen and Hankinson, 2008). Obesity during adolescence has been associated with polycystic ovary syndrome (PCOS), which is characterised by elevated levels of luteinising hormone (LH) and possibly increased risk of ovarian cancer (Schildkraut *et al*, 1996). Paradoxically, although, adiposity in premenopausal women without PCOS (Cramer *et al*, 2002) and postmenopausal women (Malacara *et al*, 2001) has been related to lower LH levels. Obesity in pre-adolescent and adolescent girls is associated with hyperinsulinemia and increased production of androgens (Stoll, 1998), which may be related to ovarian cancer risk (Risch, 1998). Also, lower levels of progesterone could increase risk (Risch, 1998). Obesity in adolescence is associated with increased risk of ovulatory infertility in adulthood (Stoll, 1998), which leads to decreased progesterone levels, and infertility has been associated with a modest increase in ovarian cancer risk (Tworoger *et al*, 2007a). Clearly, more research is needed to clarify the biological mechanisms underlying these associations.

The observed positive association between height and ovarian cancer risk is consistent with results from previous studies in several populations (Rodriguez *et al*, 2002; Engeland *et al*, 2003; Schouten *et al*, 2003, 2008). Interestingly, the positive association for height has been restricted to or stronger among younger or premenopausal women (Kuper *et al*, 2002; Lukanova *et al*, 2002; Engeland *et al*, 2003; Schouten *et al*, 2008), consistent with our findings. It is also possible that the difference in the observed association for height in the NHS and the NHSII could be due to a cohort effect.

Despite consistency across epidemiological studies, the biological mechanisms explaining the observed associations for height are unclear. Adult height may be a marker for genetic factors or of energy intake, caloric restriction, or exposure to sex and growth hormones in early life (Schouten *et al*, 2003). Height consistently has been associated with risk of other cancers (Gunnell *et al*, 2001), and the growth hormone/insulin-like growth factor (IGF) axis is a potential pathway (Gunnell, 2000). However, epidemiological studies of circulating levels of IGF and ovarian cancer risk are inconclusive (Tworoger *et al*, 2007b), and thus other mechanisms may be involved.

There are almost no epidemiological data on the relation of birthweight to risk of ovarian cancer. In a small retrospective medical record review, birthweight was not associated with mortality from ovarian cancer, but greater weight gain in the first year of life was associated with increased mortality (Barker *et al*, 1995). The investigators hypothesised that patterns of gonadotropin release are established *in utero* and during infancy, and that this could influence ovarian cancer pathogenesis later in life. A population-based case–control study found no overall association between birthweight and ovarian cancer risk, although among women younger than the age of 55 years, there was a decreased risk of ovarian cancer for those who weighed <5.5 pounds at birth compared to those who weighed 5.5–9 pounds (Rossing *et al*, 2008). Our findings do not support an association of birthweight with ovarian cancer risk, although this should be confirmed in other populations.

Our study has several limitations. Although we combined data from two large cohort studies, we had limited power to examine interactions or variation by histological type. Another limitation is the reliance on recall of body size in early life. Important strengths of our study include its large sample size, its prospective design, the confirmation of ovarian cancer cases, and the detailed information on menopausal status and ovarian cancer risk factors.

In summary, this study suggests that body fatness during childhood and adult height may be related to ovarian cancer risk, and that these associations may vary by menopausal status or age. This is the first study to examine the associations of body fatness during childhood with ovarian cancer risk, and it is one of the largest, most comprehensive studies of the other body size measures to date. Further research should confirm these findings in other populations as well as examine the underlying biological mechanisms.

## Figures and Tables

**Table 1:** Timing of body size assessments and numbers of participants, cases, and person-years included in each analysis, by study population

**Body size measure**	**Cohort^a^**	**Year assessed^b^**	**No. of participants**	**No. of cases**	**No. of person-years**
Body fatness at ages 5 and 10 years	NHS	1988	64 876	372	888 845
	NHSII	1989	110 274	131	1 614 085
Body mass index at the age of 18 years	NHS	1980	84 643	581	1 680 079
	NHSII	1989	112 119	136	1 640 636
Height	NHS	1976	110 311	735	2 528 901
	NHSII	1989	113 059	137	1 654 409
Birthweight	NHS	1992	49 321	226	521 364
	NHSII	1991	77 952	88	1 016 804

a The Nurses' Health Study (NHS) began in 1976 and had 121 700 participants at enrolment, 30–55 years of age. The Nurses' Health Study II (NHSII) began in 1989 and had 116 609 participants at enrolment, 25–42 years of age.

b The year when each body size measure was assessed defined the baseline for that analysis; follow-up was through 31 May 2004 for NHS and 31 May 2005 for NHSII.

**Table 2:** Age-standardised^a^ characteristics in 1988 (Nurses' Health Study) and 1989 (Nurses' Health Study II) by body size measures and study population

	**Average childhood body fatness^b^**		**BMI at the age of 18 years, kg m^−2^**		**Height, m**		**Birthweight, lbs**	
	**1**	**⩾5**	**<20**	**⩾25**	**<1.6**	**⩾1.75**	**<5.5**	**⩾8.5**
**Nurses' Health Study (NHS)**								
Sample size at baseline^c^	19 371	4633	29 077	8212	24 877	5165	3312	6868
								
*Means*								
Age (years)	55.4	53.9	54.4	54.6	55.0	53.8	53.6	54.9
Age at menarche (years)	12.8	12.2	12.8	12.2	12.3	12.9	12.4	12.6
Parity (among parous women)	3.2	3.0	3.1	3.1	3.2	3.0	3.1	3.1
Duration of oral contraceptive use (months)	24.6	24.0	25.2	23.2	22.9	23.2	25.9	24.7
Current body mass index (kg m^−2^)	24.4	28.1	23.4	30.8	25.7	25.1	25.5	26.1
								
*Percentages*								
Parous	93.0	92.3	92.5	90.8	92.4	90.5	92.6	92.5
Premenopausal	31.6	31.7	27.8	27.1	25.2	25.5	34.2	33.3
Postmenopausal	68.2	68.2	70.1	70.6	69.3	69.7	65.0	65.8
Family history of ovarian cancer^d^	2.6	2.8	2.5	2.9	2.2	2.7	3.0	2.6
History of tubal ligation	17.4	17.9	17.0	18.3	17.0	15.5	19.3	18.2
Current postmenopausal hormone user (among postmenopausal women)	25.3	22.3	27.8	20.9	25.2	26.8	23.4	24.8
								
**Nurses' Health Study II (NHSII)**								
Sample size at baseline^c^	19 220	6984	44 078	11 745	21 440	8922	2834	11 467
								
*Means*								
Age (years)	34.6	34.8	34.3	34.3	34.5	34.0	35.0	33.7
Age at menarche (years)	12.8	12.0	12.7	12.0	12.2	12.7	12.3	12.5
Parity (among parous women)	2.1	2.0	2.1	2.0	2.0	2.0	2.1	2.1
Duration of oral contraceptive use (months)	46.9	43.0	45.6	39.3	42.7	44.1	45.7	43.3
Current body mass index (kg m^−2^)	22.1	27.6	21.4	31.3	24.3	23.9	24.1	24.5
								
*Percentages*								
Parous	69.9	62.5	70.8	56.7	70.2	64.8	68.0	67.3
Premenopausal	99.0	98.6	99.0	98.3	98.7	99.1	98.7	98.8
Family history of ovarian cancer^d^	1.5	1.9	1.5	1.8	1.5	1.6	1.8	1.5
History of tubal ligation	16.5	16.1	15.1	14.9	17.3	13.6	17.1	14.5

a All factors except age were age-standardised in 5-year intervals for each cohort.

b Average childhood body fatness was calculated by taking the average of each participant's body fatness at ages 5 and 10 years, using a nine-level figure drawing.

c Baseline for each analysis was the year when the body size measure was assessed (see Table 1).

d Mother or sister had ovarian cancer according to the participant's response on the questionnaire; family history was evaluated using data from 1992 for NHS and 1993 for NHSII because it was not available in previous cycles.

**Table 3:** Multivariate^a^ relative risks (RR) and 95% confidence intervals (CI) of epithelial ovarian cancer according to childhood body fatness and BMI at the age of 18 years, by study population

	**Nurses' Health Study (NHS)**			**Nurses' Health Study II (NHSII)**		
	**Cases**	**Person-years**	**Multivariate^a^ RR (95% CI)**	**Cases**	**Person-years**	**Multivariate^a^** **RR (95% CI)**
*Body fatness at the age of 5 years*						
1	169	362 320	1.0 (REF)	27	397 883	1.0 (REF)
2	89	205 923	0.97 (0.75–1.26)	36	513 243	1.12 (0.68–1.85)
3	54	155 820	0.78 (0.58–1.07)	38	385 284	1.48 (0.90–2.42)
4	32	96 839	0.74 (0.50–1.08)	17	206 119	1.18 (0.64–2.17)
⩾5	28	67 943	0.87 (0.58–1.29)	13	111 557	1.65 (0.85–3.20)
*P* for trend^b^			0.11			0.12
			*P* for heterogeneity^c^=0.03			
						
*Body fatness at the age of 10 years*						
1	143	274 838	1.0 (REF)	18	301 794	1.0 (REF)
2	102	233 863	0.87 (0.67–1.13)	32	495 385	1.16 (0.65–2.08)
3	50	158 127	0.64 (0.46–0.88)	36	365 516	1.76 (1.00–3.11)
4	41	116 397	0.72 (0.51–1.02)	29	255 624	1.91 (1.06–3.45)
⩾5	36	105 620	0.69 (0.48–0.99)	16	195 766	1.30 (0.66–2.56)
*P* for trend^b^			0.01			0.12
			*P* for heterogeneity^c^=0.01			
						
*Average body fatness at the ages 5 and 10 years* d						
1	135	263 830	1.0 (REF)	15	279 639	1.0 (REF)
1.5–2	107	241 430	0.90 (0.70–1.17)	35	495 350	1.44 (0.78–2.64)
2.5–3	61	173 145	0.73 (0.54–0.99)	37	405 487	1.80 (0.98–3.28)
3.5–4.5	43	147 540	0.61 (0.43–0.86)	32	332 410	1.79 (0.97–3.32)
⩾5	26	62 901	0.81 (0.53–1.24)	12	101 199	2.09 (0.98–4.48)
*P* for trend^b^			0.04			0.10
			*P* for heterogeneity^c^=0.01			
						
*BMI at the age of 18 years, kg m* ^*−2*^						
<20	204	578 036	1.0 (REF)	38	645 713	1.0 (REF)
20–20.9	95	314 831	0.89 (0.69–1.13)	29	288 710	1.79 (1.10–2.91)
21–22.9	147	427 126	1.02 (0.82–1.26)	38	366 025	1.87 (1.19–2.95)
23–24.9	76	200 613	1.10 (0.84–1.43)	14	169 776	1.46 (0.79–2.71)
⩾25	59	159 473	1.06 (0.79–1.41)	17	170 411	1.57 (0.88–2.79)
*P* for trend^e^			0.46			0.13
			*P* for heterogeneity^c^=0.32			

a Multivariate analyses adjusted for age (continuous), parity (continuous), duration of oral contraceptive use (continuous), tubal ligation history (yes/no), and height (<1.6, 1.6 to <1.65, 1.65 to <1.7, 1.7 to <1.75, and ⩾1.75 m).

b *P* value from multivariate model with body fatness as continuous variable.

c *P* for heterogeneity by cohort assessed using the DerSimonian and Laird random effects model.

d Average childhood body fatness calculated by taking the average of each participant's body fatness at the ages 5 and 10 years.

e *P* value from multivariate model with BMI at the age of 18 years modelled as medians of categories.

**Table 4:** Multivariate^a^ relative risks (RR) and 95% confidence intervals (CI) of epithelial ovarian cancer according to childhood body fatness and BMI at the age of 18 years, by menopausal status

	**Premenopausal (NHS and NHSII)**			**Postmenopausal (NHS and NHSII)**		
	**Cases**	**Person-years**	**Multivariate^a^ RR (95% CI)**	**Cases**	**Person-years**	**Multivariate^a^ RR (95% CI)**
*Average body fatness at the ages of 5 and 10 years* b						
1	25	239 481	1.0 (REF)	123	250 962	1.0 (REF)
1.5–2	44	422 079	1.16 (0.71–1.90)	96	232 530	0.89 (0.68–1.17)
2.5–3	32	345 738	0.96 (0.57–1.64)	59	167 233	0.79 (0.58–1.08)
3.5–4.5	31	281 222	1.12 (0.65–1.90)	38	142 234	0.60 (0.42–0.87)
⩾5	13	85 055	1.38 (0.70–2.71)	24	60 158	0.85 (0.54–1.31)
*P* for trend^c^			0.92			0.09
			*P* for interaction=0.37			
						
*BMI at the age of 18 years, kg m* ^*−2*^						
<20	64	657 807	1.0 (REF)	172	446 874	1.0 (REF)
20–20.9	38	313 846	1.22 (0.81–1.83)	81	236 512	0.90 (0.69–1.18)
21–22.9	53	401 364	1.22 (0.84–1.77)	125	322 686	1.04 (0.83–1.32)
23–24.9	26	183 682	1.40 (0.88–2.21)	61	153 997	1.04 (0.78–1.40)
⩾25	26	170 607	1.41 (0.89–2.24)	43	123 277	0.91 (0.65–1.27)
*P* for trend^d^			0.10			0.85
			*P* for interaction=0.11			

a Multivariate analyses adjusted for age (continuous), parity (continuous), duration of oral contraceptive use (continuous), tubal ligation history (yes/no), and height (<1.6, 1.6 to <1.65, 1.65 to <1.7, 1.7 to <1.75, and ⩾1.75 m).

b Average childhood body fatness calculated by taking the average of each participant's body fatness at the ages 5 and 10 years.

c *P* value from multivariate model with body fatness modelled as continuous variable.

d *P* value from multivariate model with BMI at the age of 18 years modelled as medians of categories.

**Table 5:** Multivariate^a^ relative risks (RR) and 95% confidence intervals (CI) of epithelial ovarian cancer according to height and birthweight, by study population

	**Nurses' Health Study (NHS)**			**Nurses' Health Study II (NHSII)**			**NHS and NHSII combined**		
	**Cases**	**Person- years**	**Multivariate^a^ RR (95% CI)**	**Cases**	**Person-years**	**Multivariate^a^ RR (95% CI)**	**Cases**	**Person-years**	**Multivariate^a^ RR (95% CI)**
*Height, m*									
<1.6	148	572 360	1.0 (REF)	18	314 588	1.0 (REF)	166	886 961	1.0 (REF)
1.6 to <1.65	217	734 892	1.16 (0.94–1.43)	36	448 775	1.41 (0.80–2.49)	253	1 183 667	1.19 (0.98–1.45)
1.65 to <1.7	198	710 542	1.11 (0.89–1.37)	35	452 681	1.39 (0.78–2.47)	233	1 163 223	1.14 (0.93–1.39)
1.7 to <1.75	135	394 339	1.39 (1.10–1.75)	32	308 771	1.96 (1.10–3.50)	167	703 111	1.46 (1.17–1.81)
⩾1.75	37	116 768	1.27 (0.88–1.82)	16	129 593	2.35 (1.19–4.63)	53	246 361	1.43 (1.05–1.96)
*P* for trend^b^			0.01			0.01			0.001
			*P* for heterogeneity^c^=0.22						
									
*Birthweight, lbs*									
<5.5	13	34 925	0.99 (0.55–1.78)	2	36 519	0.68 (0.16–2.84)	15	71 444	0.93 (0.54–1.60)
5.5–6.9	69	165 868	1.08 (0.79–1.48)	29	303 063	1.19 (0.74–1.92)	98	468 931	1.11 (0.85–1.44)
7.0–8.4	101	248 159	1.0 (REF)	45	527 664	1.0 (REF)	146	775 823	1.0 (REF)
⩾8.5	43	72 412	1.32 (0.92–1.90)	12	149 557	0.83 (0.44–1.58)	55	221 969	1.17 (0.85–1.60)
*P* for trend^d^			0.58			0.45			0.92
			*P* for heterogeneity^c^=0.35						

a Multivariate analyses for height adjusted for age (continuous), parity (continuous), duration of oral contraceptive use (continuous), tubal ligation history (yes/no), and body mass index at the age of 18 years (<20, 20–20.9, 21–22.9, 23–24.9, and ⩾25 kg m^−2^). Multivariate analyses for birthweight adjusted for same factors except height (<1.6, 1.6 to <1.65, 1.65 to <1.7, 1.7 to <1.75, and ⩾1.75 m) instead of body mass index at the age of 18 years.

b *P* value from multivariate model with height modelled as medians of categories.

c *P* for heterogeneity by cohort assessed using the DerSimonian and Laird random effects model.

d *P* value from multivariate model with birthweight categories modelled as ordinal.
